# The additional benefit of residual spraying and insecticide-treated curtains for dengue control over current best practice in Cuba: Evaluation of disease incidence in a cluster randomized trial in a low burden setting with intensive routine control

**DOI:** 10.1371/journal.pntd.0006031

**Published:** 2017-11-08

**Authors:** Maria Eugenia Toledo, Veerle Vanlerberghe, Julio Popa Rosales, Mayelin Mirabal, Pedro Cabrera, Viviana Fonseca, Tania Gómez Padrón, Mirtha Pérez Menzies, Domingo Montada, Patrick Van der Stuyft

**Affiliations:** 1 Department of Epidemiology, Institute of Tropical Medicine “Pedro Kourí”, La Habana, Cuba; 2 Department of Public Health, Institute of Tropical Medicine, Antwerp, Belgium; 3 Provincial Center of Surveillance and Vector Control, Santiago de Cuba, Cuba; 4 Finlay Institute - Center for Vaccines Research and Production, Habana, Cuba; 5 Provincial center of Epidemiology, Santiago de Cuba, Cuba; 6 University of Ghent, Ghent, Belgium; INDEPENDENT RESEARCHER, UNITED STATES

## Abstract

**Background:**

*Aedes* control interventions are considered the cornerstone of dengue control programmes, but there is scarce evidence on their effect on disease. We set-up a cluster randomized controlled trial in Santiago de Cuba to evaluate the entomological and epidemiological effectiveness of periodical intra- and peri-domiciliary residual insecticide (deltamethrin) treatment (RIT) and long lasting insecticide treated curtains (ITC).

**Methodology/Principal findings:**

Sixty three clusters (around 250 households each) were randomly allocated to two intervention (RIT and ITC) and one control arm. Routine *Aedes* control activities (entomological surveillance, source reduction, selective adulticiding, health education) were applied in the whole study area. The outcome measures were clinical dengue case incidence and immature *Aedes* infestation. Effectiveness of tools was evaluated using a generalized linear regression model with a negative binomial link function.

Despite significant reduction in *Aedes* indices (Rate Ratio (RR) 0.54 (95%CI 0.32–0.89) in the first month after RIT, the effect faded out over time and dengue incidence was not reduced. Overall, in this setting there was no protective effect of RIT or ITC over routine in the 17months intervention period, with for house index RR of 1.16 (95%CI 0.96–1.40) and 1.25 (95%CI 1.03–1.50) and for dengue incidence RR of 1.43 (95%CI 1.08–1.90) and 0.96 (95%CI 0.72–1.28) respectively. The monthly dengue incidence rate (IR) at cluster level was best explained by epidemic periods (Incidence Rate Ratio (IRR) 5.50 (95%CI 4.14–7.31)), the IR in bordering houseblocks (IRR 1.03 (95%CI 1.02–1.04)) and the IR pre-intervention (IRR 1.02 (95%CI 1.00–1.04)).

**Conclusions:**

Adding RIT to an intensive routine *Aedes* control programme has a transient effect on the already moderate low entomological infestation levels, while ITC did not have any effect. For both interventions, we didn’t evidence impact on disease incidence. Further studies are needed to evaluate impact in settings with high *Aedes* infestation and arbovirus case load.

## Author summary

This is the first cluster randomized controlled trial providing evidence on the effect of applying residual insecticide treatment and deploying insecticide treated curtains on clinical dengue case incidence. In a context of intensive routine control activities, low *Aedes* infestation and moderate transmission, we did not observe an entomological nor epidemiological effect of these interventions. Besides the epidemiological context, we hypothesized two factors to explain our results: the counteracting effect of multiple insecticides applied in the same place at the same time and the importance of human mobility in dengue transmission dynamics. The specific impact of residual insecticide treatment and insecticide treated curtains on dengue transmission should be unambiguously demonstrated in settings with more intense arbovirus transmission and/or with less intensive routine control measures in place, before making recommendations for their implementation in local vector control programs in such contexts.

## Introduction

Dengue is a growing problem worldwide, and it is currently present in more than 100 countries [1]. Recently, chikungunya and Zika, two other *Aedes*-borne diseases, have also been spreading geographically, and they represent a growing public health threat [2, 3]. The recent co-circulation of these three *Aedes*-borne diseases in several parts of the world represents a new challenge for healthcare systems and requires the scientific world to devise effective and efficient control tools and strategies [4, 5]. Dengue control programmes have existed for decades, and they are now being extended to the control of the above-mentioned viral diseases. Routinely employed dengue vector control tools and strategies are costly, require labour-intensive delivery, have poor long-term sustainability and are failing to control local arbovirus transmission and its geographical spread [6–8]. One example of a routinely applied measure to control the adult *Aedes* mosquito during an outbreak is ultralow-volume insecticide application. However, in reality, this has a rather limited effect because lethal amounts of insecticides do not reach most indoor mosquitoes, attributable to rarely followed standardized implementation procedures [9, 10] and because mosquitoes are increasingly developing insecticide resistance [11].

While vector control interventions are currently considered the cornerstone of dengue control programmes, there is unfortunately scarce evidence on their effectiveness on dengue incidence [12]. Decision-makers of national control programmes have to formulate policies based on only a few published studies; however, owing to publication bias and non-standardized designs, these may provide inaccurate guidance.

Reliable evidence on *Aedes* control tools and strategies is needed urgently in view of the epidemiology of current *Aedes*-borne diseases and the contraindication of the use of Dengvaxia, the first dengue vaccine, for children younger than 9 years [13]. The international dengue research community has stressed the need for developing, evaluating and implementing innovative, integrated and synergistic interventions that combine the best vector control tools with recently commercialized dengue vaccines [14, 15].

The use of indoor insecticide residual spraying (IRS) is being advocated for *Aedes* control by the CDC [16] and PAHO [17], although it is unclear what IRS entails for *Aedes* control. IRS is also used for controlling other disease-transmitting vectors, making it an interesting option for integrated vector management [18]. In a recent meta-analysis on dengue vector control [12], only two observational studies evaluating this method were reported [19, 20]; unfortunately, their results regarding the impact on entomological infestation levels were contradictory. More recently, in Peru, a pilot study in 36 houses showed that deltamethrin caused *Aedes* mortality greater than 80% on treated surfaces for up to 8 weeks after IRS application [21], indicating its potential for sustained *Aedes* control. Therefore, there is a need for a controlled experimental field study, evaluating the health impact in terms of entomological and dengue incidence indicators under real-world conditions [22], to ensure the relevance of the study results for public health. The entomological effect of insecticide treated curtains (ITC) has been evaluated in several contexts [23–27], but not yet the effect on dengue incidence. As these vector control interventions are implemented in a delimited geographical area comprising several houses or house-blocks, the unit of intervention in such a trial need to be at cluster level.

We report on a cluster randomized controlled trial in which the effect of indoor and peri-domestic residual insecticide treatment (RIT) is compared to insecticide-treated curtains (ITC) and a routine *Aedes* control programme in Santiago de Cuba during epidemic and inter-epidemic periods. The setting has seasonal fluctuating low entomological infestation levels (with average House Index of 2%) and low clinical dengue incidence, with increasing outbreaks in the last decade and reaching 20 clinical cases/100 000 inhabitants over the last few years. Such settings—where *Aedes* infestation and dengue circulation are intensifying or re-emerging or, on the contrary, declining from high levels as result of intensified control efforts—are bound to become more prevalent worldwide. Within this trial, we provide evidence from such a setting and evaluate the factors influencing its effectiveness by applying Wilson et al.’s measure [22] for vector control tool studies.

## Methodology

### Study setting

The study was conducted in Santiago in southeast Cuba. *Aedes* proliferation is favoured by various factors, including the presence of an average of four water-holding containers of different types in each house [28], high population density, uncontrolled urbanization, deficient solid and liquid waste management, high temperatures (28–34°C) and rainfall from June to September. Despite an intensive routine *Aedes* control programme (ACP), *Aedes* infestation persists with an average house index (HI) of 2% for Santiago; this value may be much higher at the house-block level, which may explain the sporadic outbreaks seen since 1997 [29–31].

The standard control activities conducted by the ACP teams include entomological surveillance and source reduction through periodic inspection of houses, larviciding (with temephos) of water-holding containers, selective adulticiding (fogging with cipermethrine and clorpiriphus or perifocal residual spraying with deltamethrin) when *Aedes* foci or dengue cases are detected, providing health education, promoting community-based environmental management and enforcing mosquito control legislation through the use of fines (S1).

### Study design

We set up a cluster randomized controlled trial between April 2011 and April 2013. This study was designed to be implemented and followed-up over a 2-year period. However, owing to the major destruction of houses caused in the study area by Hurricane Sandy, this study was interrupted at the end of October 2012.

The twelve urban health areas in the Santiago municipality were included in the study. Vector control interventions are implemented at the level of geographical areas, therefore a cluster trial is indicated for the evaluation of effectiveness. Sixty-nine house-blocks were selected based on their elevated *Aedes* infestation in the previous five years. Each house-block and 3–4 of its surrounding house-blocks formed a cluster, resulting in a total of 250 houses. The 69 clusters were then ranked based on the *Aedes* infestation levels between January and December 2009, and they were mapped. During mapping, six clusters with common boundaries were excluded to control for a potential spill-over effect [32], and they were replaced by the next cluster in the ranked list. The remaining 63 clusters were randomly allocated by JPR and PVdS to the control, RIT or ITC intervention groups blocked by health area and by using the random number selection function in Excel.

The primary outcome measure (as described in the study protocol) was HI, however, the intensive dengue transmission during the study period permitted to evaluate disease incidence measures and the public health impact of the intervention.

The sample size (number of clusters) was determined using Hayes and Bennett’s calculation [33]; it had a power of 80% to detect a 50% reduction in the HI (on average, 2%) at an alpha error level of 0.05 (assuming a between-cluster coefficient of variation of 0.5). We increased the number of clusters by 5%, resulting in a final sample size of 21 clusters of 250 houses/study arm. The study showed 80% power to detect a 50% reduction in the dengue incidence of 10 cases/10 000 inhabitants.

### Interventions

To prepare for implementing the trial, two workshops were held with the health area medical team, vector control team and formal and informal community leaders to explain the set-up and objective of the trial. Separate meetings were held with the population of the selected clusters, as described below.

In all arms, routine control activities were continued. In the control arm, this was the only strategy.

#### Residual insecticide treatment (RIT)

Before the first application, several meetings were conducted to inform the target population, and community consent was obtained. Individual households had the choice to refuse the application at each moment. ACP teams were trained to perform this spraying activity, and standard operating procedures were designed. Before each application round, ACP workers were retrained with the support of a short video showing the correct application procedures. Quality control of the insecticide dilution and application was performed on a sampling basis by the supervisors.

K-Othrine 25 WG, supplied by Bayer Environmental Sciences Co. (25% deltamethrin granular formulation to be dissolved in water, 20 g in 8 L), was sprayed every four months (three times/year) in dosages of 20–25 mg a.i./m^2^, with an expected residual effect of 3–6 months, as recommended by World Health Organization Pesticide Evaluation Scheme (WHOPES) for indoor residual spray [34]. A total of five treatment cycles were realized during the whole study period: mid-April to mid-May 2011, September 2011, mid-January to mid-February 2012, May to June 2012 and October 2012. Inspired by the targeted spraying for *Aedes* described by Ritchie *et al*. [35] and the preferential resting behaviour of the *Aedes* mosquitoes [36], the RIT consisted of insecticide application on surfaces where mosquitoes usually rest inside the house, including the lower half of walls, underside of beds, kitchen sink and furniture, back side of doors (especially those of bathrooms), behind refrigerators and inside closets. Additionally, in the houses (but not in public premises), the insecticide was also applied intra- and peri-domestically on the external surface of (ground-level) water tanks and surrounding wall areas, surface behind the tank and a 50-cm area on both sides of and above the tank.

#### Insecticide-treated curtain (ITC)

Before distribution, group discussions were held with the population of the selected communities to explain and discuss the study purpose and use of the ITC. We handed out information leaflets, and one member of each family received detailed person-to-person instructions on the use and maintenance of the ITC. The ITC was made from PermaNet polyester netting (Vestergaard-Frandsen, Switzerland) treated with a long-lasting formulation of deltamethrin (55 mg/m^2^) and coated with a protectant (no details disclosed by the manufacturer) to prevent the degradation of the insecticide when exposed to UV light. The ITC retains its insecticidal properties and efficacy for around 2 years (information from producer). All curtains have white patterned netting and a size of 1.1 m (width) × 2.9 m (height) (people could modify this according to their needs). All households were revisited a few days later to obtain informed consent from the head of the household and negotiate where the curtains would be placed. The technical criteria for selecting places to hang the ITC (optimal sites from an entomological perspective) were reconciled with family preferences. The families generally perceived that the bedroom and living room areas had the most mosquitoes, and they preferred to hang the ITCs by windows or door openings or on the wall. At most three ITCs were offered, which was equal to the number of rooms in a typical house. In schools, working areas and public premises, more curtains were distributed depending on the number of rooms/windows.

Curtains damaged during use were replaced. If new persons came to live in the house-blocks during the study, they were asked if they were willing to be included in the study.

### Data collection and analysis

#### RIT implementation coverage

The number of premises sprayed was obtained from signed-off application forms that were filled by the ACP teams during each application round.

#### ITC uptake

At the time of distribution, we recorded the number of ITCs distributed in each household in the intervention cluster. The continued use of curtains was promoted during subsequent routine household visits by ACP workers. In October 2011, the use of ITC was recorded in all houses. The number of targeted structures (wall, doors and windows) was not recorded, as these, being structures with very different surfaces, together with the number of ITCs distributed, would not provide information on the percentage of the total surface covered.

#### *Aedes* susceptibility to deltamethrin

Larvae detected in water-holding containers by routine ACP visits in March 2010 were reared to adults in the entomological laboratory of the Institute of Tropical Medicine ‘Pedro Kourí’ in Havana. Non-blood fed female mosquitoes of age 3–5 days were screened for deltamethrin susceptibility using the standard WHO tube bioassay protocol [37, 38]. The *Aedes* Rockefeller strain, a susceptible laboratory strain of Caribbean origin, was used as a reference. Four bioassays were performed with five replicates of 25 female mosquitoes per bioassay: four replicates were exposed to insecticide-treated paper, and one control replicate was exposed to untreated paper. We calculated the proportion of local *Aedes* strain mosquitoes that died at 24 h out of the total number exposed. Tests with more than 20% control mortality, if any, were discarded and repeated. When the control mortality was 5%–20%, the mortality was corrected using Abbot’s formula [39].

#### Deltamethrin bioavailability

Six months after distribution, we tested the deltamethrin bioavailability in 15 randomly collected ITCs: eight were never washed, five were washed once and two were washed twice, with two untreated curtains serving as controls. Tube bioassays were conducted at 25 ± 2°C and 75 ± 10% relative humidity by using five tubes per curtain following the standard WHO procedure [40]. Ten female mosquitoes were introduced into each tube, which remained vertical throughout the bioassay, and exposed to the ITC sample for 3 min. We calculated the average mosquito mortality per curtain and within curtain groups (unwashed, washed one time and washed two times). If the control mosquito mortality was above 5%, the results were discarded and the assays were repeated. The national pesticide analytical laboratory (Instituto de Investigaciones de Sanidad Vegetal, Havana) determined the deltamethrin concentration from four ITCs taken from four randomly selected houses using high-performance liquid chromatography (HPLC) following standardized procedures [41].

After the RIT application of April 2011 and January 2012, we tested the deltamethrin bioavailability on different surfaces (cement, unpainted wood and metal) in four randomly selected houses 1, 2 and 3 weeks, 1 and 3 months after application. Cone bioassays were conducted using two cones per surface type (cement, unpainted wood and metal) following the standard WHO procedure [40]. Twenty female mosquitoes (age 3–5 days, non-blood-fed) were introduced into each cone, which remained attached to the surface for 1 h. We calculated the average mosquito mortality per surface type and per exposure time. An assay with the same procedure was applied in a neighbouring untreated house outside the intervention clusters under the same weather conditions to serve as a control. If the control mosquito mortality was above 5%, the results were discarded and the assays were repeated.

#### Entomological impact

Routine ACP house inspections are conducted once a month in all dwellings of the municipality. For all immature stages found, the species is identified. We extracted from the routine ACP records for the Santiago municipality as a whole and for each study cluster, information on the number of houses with at least one container positive for immature *Aedes* stages and with at least one container positive for *Aedes* pupae collected per house-block from March 2011 to October 2012 (from 2 months before up to 17 months after the start of the intervention). The number of houses per cluster was extracted from these records, and the population was calculated based on an estimate with an average of four inhabitants/house in the urban Santiago municipality.

*Aedes* infestation levels were the secondary trial outcome. We calculated HIs and pupal index (PI) (/1 000 persons) per study cluster per month. We averaged the entomological indicators over the 2-month period before the start of the intervention to obtain baseline infestation levels per study arm. We analysed the intervention effect over the 17-month period starting from June 2011, the month of the first house inspection cycle after RIT and ITC implementation. For descriptive purposes, we graphed the average monthly HI per trimester (and its standard error) for each study arm. To evaluate the effect of the intervention, we constructed generalized linear random effect regression models with a negative binomial link function. We evaluated the effect (RIT or ITC versus control clusters) on the monthly cluster-level HI and PI. This model considered the nature of the data (repeated measurements and cluster design). For the RIT study arm, we also evaluated the effect of RIT on *Aedes* infestation by month after each application by considering four treatment cycles. We estimated the difference of differences (and 95% CI) between RIT and control clusters by using the same model.

#### Epidemiological impact

The dengue incidence rate (/10 000 persons/month) was the primary trial outcome. The municipal epidemiology department collects surveillance data on disease occurrence. A clinical case of dengue is defined as a suspect case (fever and one of the following signs or symptoms: myalgia, arthralgia, retro orbital pain, headache or rash). All suspect cases are admitted to temporary, dedicated wards in a hospital and treated following a standardized protocol. On the 6th day after symptom onset, anti-dengue IgM was determined at the Provincial Centre for Hygiene and Epidemiology of Santiago (Ultra Micro ELISA). Positive samples were confirmed in the international reference laboratory of the Institute of Tropical Medicine ‘Pedro Kouri’ (IgM ELISA) [30]. The Cuban dengue surveillance system records all febrile cases presenting themselves in health centres and hospitals, and it actively screens the population for fever during outbreaks.

We extracted information on the number of confirmed (as per national protocol explained above) dengue cases per cluster and per month from these surveillance records for the period from January 2010 to October 2012 (from 16 months before up to 17 months after the start of intervention) for the Santiago municipality as a whole and for each study cluster.

To control for epidemiological pressure from the zones bordering the study clusters, we extracted epidemiological information on the number of confirmed dengue cases from the bordering house-blocks (surrounding the study clusters) per month over the same period.

The dengue incidence rate (/10 000 persons/month) was calculated per study cluster and per month. The incidence rate for study clusters and bordering house-blocks (with 95% CI) was calculated for the pre-intervention period as a whole, separately for epidemic months (defined as more than 1 case/10 000 inhabitants/month in the Santiago municipality) and for non-epidemic months. We analysed the intervention effect over the 17-month period starting from June 2011, the month of the first house inspection cycle after the start of the RIT and ITC interventions. We constructed generalized linear random effect regression models with a negative binomial link function by considering the nature of the data (repeated measurements and cluster design). We evaluated the effect (RIT or ITC versus control clusters) on the monthly cluster-level dengue incidence rate. We adjusted for confounding by the epidemic period, pre-intervention incidence rate and/or incidence rate in bordering house-blocks per reporting period. The Akaike information criterion (AIC) was computed for each model, and the lowest AIC was taken to indicate the best fitting model.

We used SPSS 23.0, Stata 10.0 and R statistical software for the analysis.

### Ethical considerations

This study was approved by the ethical committee of the Institute of Tropical Medicine ‘Pedro Kourí’, national health authorities, Institutional Review Board of the Institute of Tropical Medicine and University of Antwerp (Belgian registration no. B300201111923). During a meeting in each study cluster with community members after randomization, but before the start of the intervention, community approval was obtained. Written informed consent was obtained from the head of every household (18 years or older) included in the ITC arm. Residual spraying, as described in the study setting section, is occasionally implemented as a routine activity by the control programme. Therefore, with approval of the ethics committees, the heads of households (18 years or older) in the RIT arm signed at each application the ACP worker’s activity report form as proof of consent, instead of a separate form. The RIT and ITC used/applied insecticides approved by the WHOPES. The trial was registered at the Current Controlled Trials register (no. ISRCTN27037293). The RIT insecticide and ITC were purchased from the study budget and freely applied/distributed to the population. The study was conducted in accordance with the Helsinki Declaration of 1964 and subsequent revisions.

## Results

**Fig 1:**
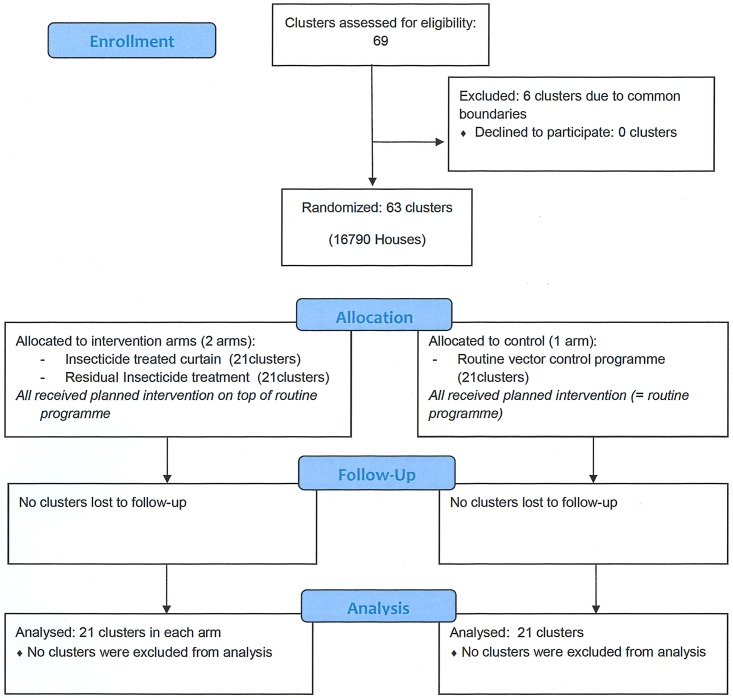
CONSORT flowchart.

The 63 clusters (21 per study arm) comprised 16 790 houses (S1 Map). All clusters completed the study protocol from April 2011 up to the end of October 2012, and all were included in the analysis (Fig 1). The pre-intervention entomological infestation levels and epidemiological characteristics were comparable in the three study arms (Table 1). The local pre-intervention (2010) strain of *Aedes* mosquitoes showed 100% mortality after 1 h of exposure to 0.05% deltamethrin (400 mosquitoes exposed).

**Table 1:** Baseline characteristics of the study clusters, Santiago, Cuba, 2010–2011.

	Intervention arms		Control arm
	ITC	RIT	
Number of clusters	21	21	21
**Demographic characteristics**			
Population	22552	20720	23888
Range (per cluster)	484–1576	380–1968	836–1928
Mean (SD) per cluster	1073.9(337.5)	986.7(352.8)	1137.5(264.2)
Number of houses	5638	5180	5972
Range (per cluster)	120–394	95–492	209–482
Mean (SD) per cluster	267.5(84.3)	247.1(88.0)	284.4(66.0)
Entomological indicators*			
House index (%, 95%CI)	1.21 (0.70–1.73)	1.21(0.18–2.23)	0.86 (0.50–1.22)
Pupal index (/1000 inhabitants, 95%CI)	1.45 (0.29–2.61)	1.40 (0.14–2.66)	1.60 (0.45–2.7)
Epidemiological indicators**			
Dengue incidence rate (/10 000 person/month)			
Total period**			
Study clusters (95%CI)	3.99 (1.78–8.30)	3.80 (2.08–6.64)	5.47 (3.25–8.89)
Bordering houseblocks (95%CI)	4.49 (2.36–8.14)	4.04 (2.07–7.47)	4.81 (2.76–8.09)
Epidemic period***			
Study clusters (95%CI)	7.54 (3.11–16.58)	8.93 (5.04–15.15)	10.95 (6.21–18.50)
Bordering houseblocks (95%CI)	8.76 (4.45–16.24)	9.39 (4.75–17.47)	9.51 (5.18–16.62)
*Endemic period*			
Study clusters (95%CI)	1.86 (0.40–6.83)	0.72 (0.29–1.67)	2.18 (0.82–5.22)
Bordering houseblocks (95%CI)	1.93 (0.65–5.16)	0.83 (0.28–2.15)	2.00 (0.65–35.35)

* march-april 2011

** january 2010-april 2011

*** july 2010-december 2010

The RIT coverage over the five treatment cycles was, on average, 5 033 out of 5 180 premises (97.2%; range: 5 016–5 063 per round) [42]. A total of 12 937 ITCs were distributed, with an average of 2.3 ITC/house. Of the 5 617 households in the ITC clusters, 94.4% chose to receive and hang the ITC in their house, and less than 5% removed them 6 months after distribution. No important harms or unintended effects were reported in the intervention arms.

**Fig 2:**
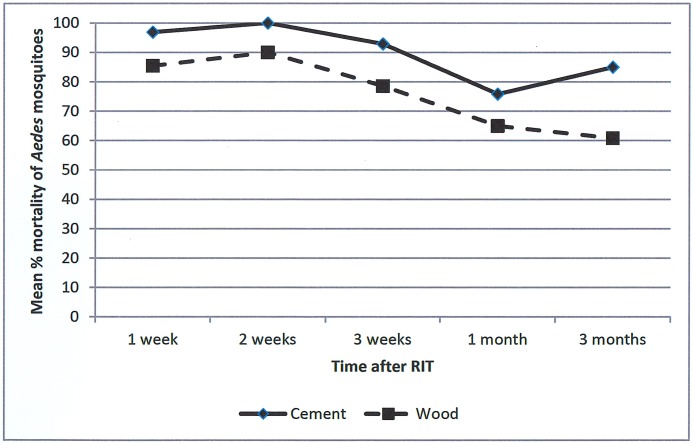
Deltamethrin bioavailability results after RIT application on different materials, 2011–2012, Santiago de Cuba.

The bioavailability of deltamethrin was better when sprayed on cement walls than on unpainted wooden materials, and it remained high (85.0%) for up to 3 months after application (Fig 2), on metal it declined quickly up to below 50% after 3 months. The bioavailability of the insecticide in the ITC after 6 months of use was good: the mortality of mosquitoes exposed to ITC that was never washed, washed one time and washed two times was 96.7%, 90.4% and 87.5%, respectively. The HPLC gave a range of 29–120 mg of active ingredient/m^2^, which is above the minimum effective concentration of 25 mg/m^2^ [43].

**Fig 3:**
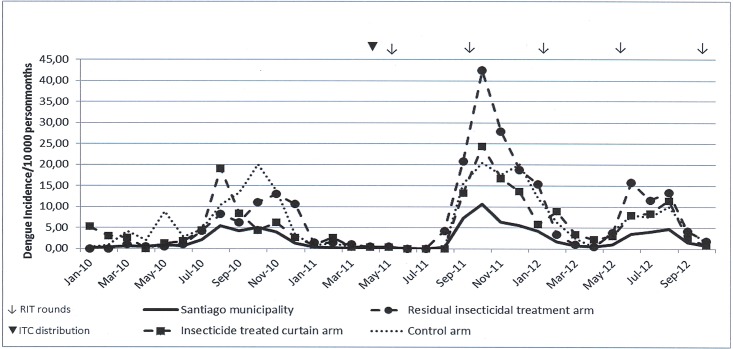
Dengue case incidence rate in study clusters and Santiago municipality, January 2010–October 2012, Santiago, Cuba.

**Fig 4:**
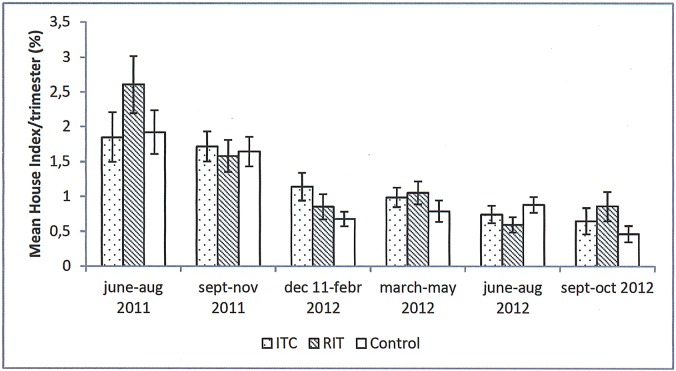
House Index (Mean % and Standard Error) in study clusters during intervention period, June 2011–October 2012, Santiago, Cuba.

Before the intervention, the HI (March 2011) was 1.21%, 1.21% and 0.86% for the RIT, ITC and control arms, respectively (Table 1), and the dengue incidence rate (January 2010 to March 2011) was 3.8, 4.0 and 5.5/10 000 persons/month, respectively. Fig 3 shows that the dengue case incidence rate in the study arms was higher than in the Santiago municipality as a whole, as was expected owing to the selection of study sites with among the highest levels of entomological infestation in the city. After the interventions were started, the usual seasonal increase in dengue case incidence can be observed in all study arms over the entire intervention period. Both RIT and ITC did not show a significant protective effect on the entomological infestation level (Fig 4) or dengue case incidence rate (Table 2); on the contrary, slight negative effects were observed for ITC on HI (rate ratio: 1.2, 95% CI (1.0–1.5)) and for RIT on dengue incidence (rate ratio: 1.4, 95% CI (1.1–1.9)). As RIT is known to have a temporary effect, we evaluated the effect of RIT by the number of months post-application over two of the four rounds (Table 3). In the first month after application, the PI reduced by 46% and the HI, by 37%. The effect faded out over time. No significant temporal effect was observed on the dengue incidence rate: average IR was 10.1 (95% CI 4.6–15.6), 13.7 (95% CI 9.5–18.0), 10.5 (95% CI 7.6–13.4) and 7.8 (95% CI 4.4–11.2) per 10.000 inhabitants, in the month of RIT application and one, two and three months after application respectively. When searching for the best-fitting model and adjusting for potential residual confounders, the negative crude effect of intervention disappeared. In the model (Table 4) with the lowest AIC, the dengue case incidence rate by cluster and month was best explained by the epidemic period (IRR = 5.50), incidence rate in bordering house-blocks (IRR = 1.03) and incidence rate before the intervention (IRR = 1.02); the study arm was not withheld in this model as an independent determinant.

**Table 2:** Effect* of insecticide treated curtain implementation and of residual insecticide treatment on *Aedes* infestation and dengue case incidence, Santiago, Cuba, 2011–2012.

	Insecticide Treated Curtains			Residual Insecticide Treatment		
	Crude Rate Ratio Intervention:Control	95% CI	P value	Crude Rate Ratio Intervention:Control	95% CI	P value
Dengue case Incidence	0.96	0.72–1.28	0.784	1.43	1.08–1.90	0.012
Pupal Index	1.15	0.94–1.41	0.163	1.14	0.93–1.40	0.204
House Index	1.25	1.03–1.50	0.022	1.16	0.96–1.40	0.134

*estimated with a negative binomial regression model taking into account repeated measurement and cluster design

**Table 3:** Effect* of residual insecticide treatment (RIT) on *Aedes* infestation by month after each application**, Santiago, Cuba, 2011–2012.

	Pupal Index			House Index		
	Rate ratio	95% CI	P value	Rate ratio	95% CI	P value
Month of RIT	1	-	-	1	-	-
Month of RIT + 1	0.54	0.32–0.89	0.017	0.73	0.51–1.03	0.074
Month of RIT + 2	0.89	0.53–1.49	0.668	1.04	0.73–1.49	0.812
Month of RIT + 3	0.87	0.53–1.43	0.585	0.92	0.65–1.30	0.647

*difference of differences between RIT and control clusters, estimated with a negative binomial regression model adjusted for repeated measurement and cluster design

**4 treatment cycles in the period April 2011-October 2012

**Table 4:** Comparison of models for dengue case incidence within study clusters controlling for potential residual confounding, Santiago, Cuba, 2010–2012.

	Crude IRR (95%CI)	Adjusted IRR **(95%CI)		
		Model 1	Model 2	Model 3
**Epidemic period**	9.54 (7.18–12.69)	5.47 (4.12–7.28)	5.50 (4.14–7.31)	5.50 (4.14–7.31)
Incidence in bordering houseblocks*	1.05 (1.04–1.06)	1.03 (1.02–1.04)	1.03 (1.02–1.04)	1.03 (1.02–1.04)
Cumulative incidence pre-intervention period*	1.03 (1.00–1.06)	-	1.02 (1.00–1.04)	-
**Study arm**				
**ITC**	0.96 (0.72–1.28)	-	-	1.01 (0.79–1.30)
**RIT**	1.43 (1.08–1.90)	-	-	1.24 (0.97–1.59)
***AIC***	-	*2284*.*6*	*2283*.*0*	*2285*.*1*
***Log likelihood***	-	*-1137*.*28*	*-1135*.*51*	*-1135*.*54*

* per 10 000 person months

** negative binomial regression model taking into account repeated measurement and cluster design

## Discussion

Residual insecticidal treatment significantly reduced the *Stegomyia* indices up to 1 month after application, but this effect was not sustained and, in our setting, it did not result in a reduction of clinical case incidence.

Adding ITC to the routine *Aedes* control programme in Cuba had no impact on the incidence of clinical dengue cases, did not substantially reduce entomological infestation levels and did not mitigate seasonal dengue outbreaks.

The design of this study is its major strength: randomized controlled trial testing with standardized interventions in 21 clusters of around 250 houses in each intervention arm (sufficient power to detect a 50% reduction in HI and dengue incidence) with a follow-up period of 17 months and the combination of entomological and epidemiological indicators to evaluate the intervention effects. According to a recent systematic review, the latter is hardly encountered in published studies evaluating the impact of dengue vector control interventions [12].

A relative weakness is that the outcome measures were extracted from routine surveillance data. This could have resulted in non-differential underreporting [44] and overall dilution of effects. Underreporting is expected to be limited to entomology data, as training for onsite inspection and for immature *Aedes* data collection were conducted for the vector campaign workers before the study; furthermore, the provincial quality control team reinforced the quality control system and intensified monitoring over the entire study period. For collecting data on dengue cases, the routine Cuban surveillance system combines a passive approach with active case finding from the moment the first dengue case is confirmed [31]. Underreporting of symptomatic cases is thus unlikely to be substantial. The secondary outcomes selected were *Aedes* immature indices because monitoring adult indices is faced with low reproducibility [45]. At any rate, for both proxies the relationship with transmission remains unclear [46], hence we relied more on the evaluation of the impact on dengue disease incidence. On the other hand, the availability of information from the entire study clusters (and beyond) and with fine-grained monthly repeated measurements for entomology enabled the analysis to be adjusted for probable confounding factors and small-scale temporal variability.

The internal validity of the study design seems secured. However, it is necessary to consider whether the observed low or short-lived entomological effect could be explained by other factors. The susceptibility of the local *Aedes* strain to deltamethrin was 100% at baseline. Until the start of this study, this insecticide was not used in the Santiago municipality, and no resistance would have developed. Resistance to deltamethrin can emerge after six months of intensive use, as seen in an outbreak in Brazil [47]. However, in our trial, deltamethrin was not used in the entire city. Instead, it was used in only two trial arms consisting of 42 clusters, and if the observed overall lack of effect is due to the development of resistance, we expect to observe an effect in at least the first 6 months of study that then wanes over time, which was not the case.

To ensure optimal implementation, which is especially important for RIT, standard operating procedures were adhered to and quality control procedures were established. In order to produce a long-term preventive effect regularity of treatment cycles was respected in line with the residual efficacy reported by the manufacturer. The bioavailability of insecticide after RIT was similar to findings reported in a study in Peru [21], with mosquito mortalities of 83% versus 76% on brick/cement and of 60% versus 64% on unpainted wood 3 months after application, in our and Peru study, respectively. The transient effect on entomological indicators after RIT application is in line with the results in the Peru study [21], although, in contrast, we could demonstrate a significant effect on immature indices in the first month after application. The bioavailability of deltamethrin on ITC after 6 months was satisfactory, but slightly lower than observed in Thailand [48].

Both RIT and ITC implementation had excellent coverage and remained at much higher levels over time than was observed in other settings [49]. RIT was well accepted, as it did not differ much from the vector control measures routinely applied during outbreaks. The ITC, a new tool for the inhabitants of the Santiago municipality, was introduced after consultation and information rounds and with the active involvement of household members.

As the clusters did not border each other, we do not expect a ‘no difference’ finding to be due to a spill-over effect, as reported in an *Aedes* control study using ITC [32]. To control for possible epidemiological pressure from surrounding areas on dengue incidence within the treatment clusters, we included the dengue incidence in house-blocks bordering the study clusters as a variable in the multivariable analysis. However, this did not lead to changes in our effect estimates.

The dissociation between entomological and epidemiological impacts could be due to the settings, with already low infestation levels or not attaining the (unknown) level to which entomological infestation has to be (sustainably?) reduced to stop or reduce transmission.

Because dengue is transmitted by mosquito bites during the day, daytime human mobility is an important factor affecting dengue transmission dynamics [50]. Differential or non-differential mobility between study arms could have diluted the intervention effect. There are no arguments for the former. As for the latter, if local effects were indeed greatly diluted, then schools, workplaces, markets and mobility hubs outside the study clusters must also be covered in addition to the living and working places within the clusters.

The lack of demonstration of an intervention effect could also be explained by the existence of an already intensive routine programme with the differential intensity of control actions over time and place in the function of the epidemiological situation and events. In addition to the inter-sector and community-based actions, the programme is heavily insecticide based with frequent indoor and outdoor fogging. In another Cuban municipality, ITC implementation also did not result in a reduction in entomological infestation [25], whereas community-based environmental management without an increase in insecticide use above routine programme levels showed a clear effect [31, 51]. This contrasts with the coverage-dependent ITC effect observed in Venezuela and Central Thailand [23, 24] and the demonstration of a significant protective effect of window screens on *Aedes* infestation [52] in settings with higher infestation levels and less-intensive routine control programmes. In contrast to the findings of this trial, IRS, an approach similar to RIT used in this trial, was estimated to reduce dengue incidence by 64% (OR: 0.36, 95% CI (0.14–0.88)) in an observational study [19]. However, Ko *et al*. [20] obtained similar results as in our study and could not demonstrate the effect of IRS on dengue incidence (OR: 1.15, 95% CI (0.63–2.10)).

In this setting, multiple insecticides were used at the same place at the same time (routine programme and study interventions), and this could have counteracting effects, as was already suggested when no benefit resulted from the combination of insecticide treated bed-nets with IRS or with a durable impregnated lining for malaria control [53, 54]. Studies on the interactions between different insecticide-based vector control efforts are urgently needed to clarify the possible mechanisms involved.

As a dengue vaccine is now available and is being rolled-out, but given its incomplete effectiveness, it is necessary to combine it with the best vector control options available to produce a synergistic effect on dengue incidence [55]. This contrasts to the control of urban yellow fever outbreaks by immunization [56], because the vaccine for the same showed much higher efficacy [57]. Furthermore, many countries currently have to address the burden of three circulating arboviruses, namely, dengue, chikungunya, and Zika, and they cannot base their control efforts on the dengue vaccine alone.

The lack of an observed effect of RIT and ITC on dengue incidence in the present cluster randomized controlled trial should perhaps not be very surprising. In a recent meta-analysis, none of the included randomized studies evaluating dengue adult vector control tools demonstrated a significant impact on dengue incidence, in contrast to other studies with less robust study designs that demonstrated an impact on dengue transmission with the intervention of house screening, IRS and environmental management [12]. In view of the high cost of RIT and ITC implementation [42, 58] and the poor effect on dengue transmission in a low incidence setting shown in this study, the effectiveness should be evaluated in an array of contexts with higher case load and a different set-up of a routine control program.

While RIT must be urgently evaluated further with robust study designs to provide more evidence on its potential effectiveness for reducing dengue transmission, a paradigm shift may also be needed. This includes evaluating/monitoring daytime human mobility in future studies [19, 50] or covering premises where most household members work, go to school or spend a substantial part of their day in geographical units of intervention, which makes a cluster randomized trial difficult to design and implement. The complexity of the dengue transmission dynamics forces us to also think out of the box. Furthermore, for routine control programmes, instead of aiming to cover entire municipalities with undifferentiated vector control efforts or focusing on the reactive implementation of insecticide-based interventions in response to clinically apparent disease manifestations, it is necessary to shift to a new strategy by using risk stratification to concentrate proactive, sustained efforts in areas at high risk for transmission [59].

Additionally, several questions remain to be addressed concerning the timing of residual adulticide application for dengue control and outcome assessment: Would application be more effective if performed proactively when an increase in caseload or an epidemic of arboviral disease is forecasted? Could it delay the start of an epidemic wave, so that time is given to prepare more efficient and comprehensive vector control measures and health system responses? Because an effective routine control program (with frequent indoor and outdoor fogging) and a limited amount of dengue (possibly due to this control program) existed in the study area, it is possible that RIT and ITC added little if any additional benefit. Therefore, before making recommendations about RIT and/or ITC during epidemics and in endemic settings with more intense arbovirus transmission and/or with less intensive routine control measures in place, the impact of these control measures on dengue transmission in these settings should be demonstrated.

## Supporting information

S1 Text Full description of the standard control activities. (DOCX)

S1 Map Map of the study site and clusters. (DOCX)

S1 Checklist CONSORT checklist. (DOCX)

S1 Protocol Study protocol. (DOCX)
