# Supplementary material for: The additional benefit of residual spraying and insecticide-treated curtains for dengue control over current best practice in Cuba: Evaluation of disease incidence in a cluster randomized trial in a low burden setting with intensive routine control
Source: PLoS Negl Trop Dis. 2017 Nov 8;11(11):e0006031. doi: 10.1371/journal.pntd.0006031 (PMC5695847; doi:10.1371/journal.pntd.0006031)
Supplement: S1 Text — (DOCX) [file pntd.0006031.s001.docx]

Supplementary material 1. Full description of the standard control activities, adapted from Baly et al. 2012 (1)

The Cuban government accords the highest priority to *Aedes* control and dengue prevention and assigns the necessary resources to conduct a wide range of activities that are intensified during epidemics that are coordinated by governmental intersectoral bodies.

The *A. aegypti* control programme is centrally regulated by the MOH but operationally decentralized to the municipal and health area level. Dedicated vector control workers perform the main programme activities: entomological surveillance, source reduction through periodic inspection of houses, larviciding with temephos in waterstorage containers, selective perifocal insecticide spraying against adult mosquitoes, health education and the enforcement of mosquito-control legislation through the use of fines. During dengue outbreaks, house inspection cycles are shortened, intra-domiciliary adulticiding (every 7 days or less) is introduced in all houses in neighbourhoods with transmission and specialized teams from provincial and national level conduct spatial spraying and reinforce quality control activities.

The polyclinics and family practices are the entry point for routine, passive, clinical-epidemiological surveillance of fever of unknown origin. When dengue transmission is suspected, active population screening for fever cases is organized with family doctors, nurses, non-health personnel from different government agencies and community organizations. All households are visited daily, and persons with fever are referred to the polyclinics and assessed. If dengue fever is suspected, they are admitted to temporary, dedicated wards in a hospital and treated following a standardized protocol. On the 6th day after symptom onset, anti-dengue IgM is determined at the Provincial Centre for Hygiene and Epidemiology of Santiago. Positive samples are reassessed in the laboratories of the Institute of Tropical Medicine ‘Pedro Kouri’ for confirmation as long as an outbreak has not been declared.

1. Baly A, Toledo ME, Rodriguez K, Benitez JR, Rodriguez M, Boelaert M, et al. Costs of dengue prevention and incremental cost of dengue outbreak control in Guantanamo, Cuba. Trop Med Int Health. 2012;17(1):123-32.
