# Supplementary material for: The additional benefit of residual spraying and insecticide-treated curtains for dengue control over current best practice in Cuba: Evaluation of disease incidence in a cluster randomized trial in a low burden setting with intensive routine control
Source: PLoS Negl Trop Dis. 2017 Nov 8;11(11):e0006031. doi: 10.1371/journal.pntd.0006031 (PMC5695847; doi:10.1371/journal.pntd.0006031)
Supplement: S1 Map — (DOCX) [file pntd.0006031.s002.docx]

Supplementary material 2. Map of the study site and clusters

Santiago de Cuba, 20°01'14.99" N -75°49'36.01" W


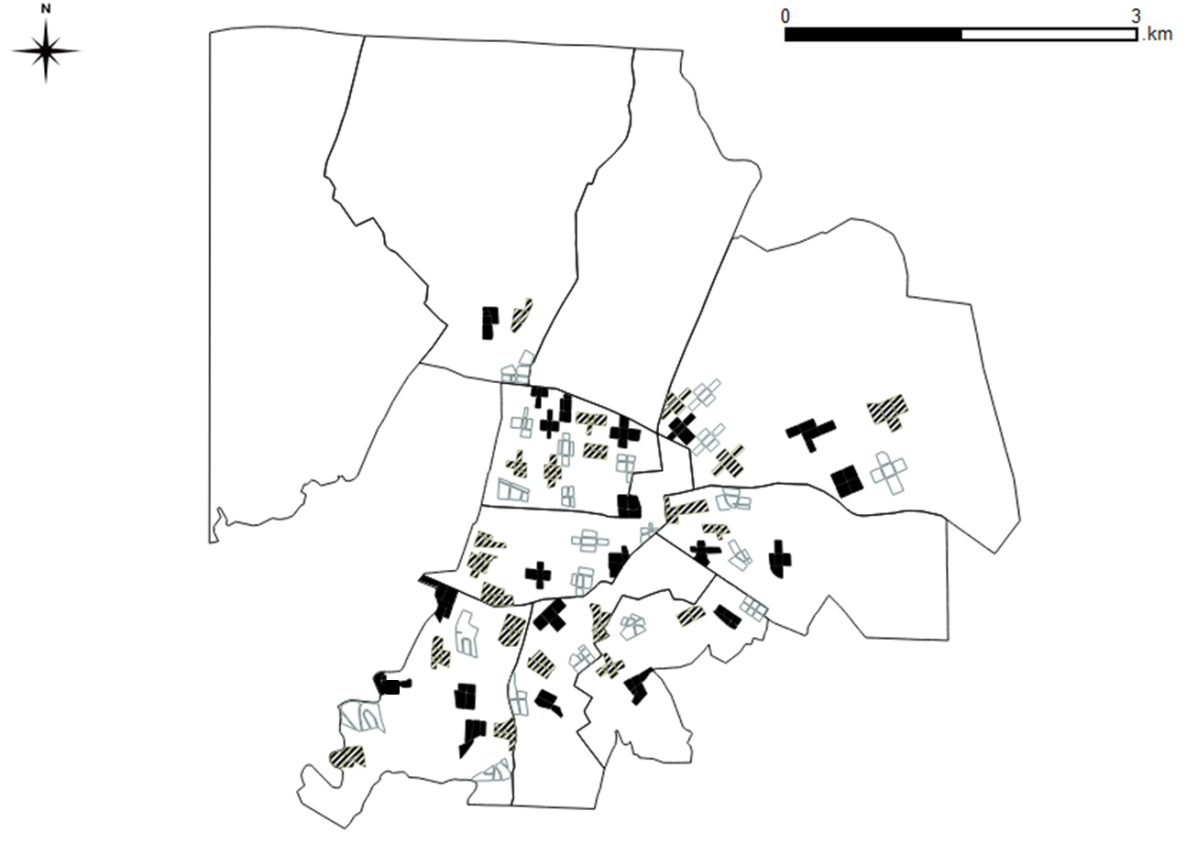


| **Legend** |  |
| --- | --- |
| Clusters RIT intervention arm |  |
| Clusters ITC intervention arm |  |
| Clusters control arm |  |
