# Supplementary material for: The additional benefit of residual spraying and insecticide-treated curtains for dengue control over current best practice in Cuba: Evaluation of disease incidence in a cluster randomized trial in a low burden setting with intensive routine control
Source: PLoS Negl Trop Dis. 2017 Nov 8;11(11):e0006031. doi: 10.1371/journal.pntd.0006031 (PMC5695847; doi:10.1371/journal.pntd.0006031)
Supplement: S1 Protocol — (DOCX) [file pntd.0006031.s004.docx]

# PROTOCOL

# I. Generalities

## *A. Title project :* Effectiveness and Cost-effectiveness of perifocal residual deltamethrin spraying and of long lasting deltamethrin treated curtains distribution for Aedes control

## *B. Collaborating institutes and investigators:*

Principal Investigators: Maria Eugenia Toledo, IPK and Patrick Van der Stuyft, ITG

Collaborators:

- ITG: Veerle Vanlerberghe, Pierre Lefèvre
- IPK: Alberto Baly, Domingo Montada, Dennis Perez
- National Vector Control programme: Juan R. Vazquez Canga, Ms. Alina
- Programa provincial de Control de Vectores, Santiago de Cuba: Julio Popa Rosales, Pedro Cabrera, Enrique Ceballo, Francisco Fabre, Mirta Perez Menzies, Rosa Maria Castillo, Lino, Karelia Gonzalez Coello

# II. Introduction and problem statement

Dengue is a viral vector-borne disease, predominantly affecting the urban areas but extending into more populated rural areas. The main insect vector is the highly synanthropic *Aedes aegypti* mosquito. The vector breeds in water storage containers and other deposits containing relative clean water, and bites and rests in and around human dwellings. Attempts at eradication failed during the 1960s in Latin America and today more than 50% of houses are infested with *Aedes* larvae in many endemic areas (WHO HQ 2000;PAHO 1994).

Dengue prevention has long depended on control of immature vector populations either by insecticidal treatment (larviciding) or removal of breeding sites and occasionally, biological control. These interventions showed a variable success and are in most of the countries difficult to sustain.

Control of immature stages impacts only on vector densities and not on mosquito longevity or other key parameters affecting dengue transmission: targeting adult vectors is more likely to reduce both transmission and mosquito density. The WHO Dengue Scientific Working Group of 2006 identified the development and evaluation of Insecticide-treated materials (ITM) as a primary global research stream. ITM, highly effective in preventing malaria and other nocturnally transmitted vector-borne diseases, have shown a first proof of efficacy on dengue vector densities and potentially on dengue transmission in Venezuela and Mexico (Kroeger et al. 2006). In a recent study in Thailand and Venezuela, it was observed that the use of the ITM curtains declined rapidly over time (Vanlerberghe et al. 2011a) and its effect was shown to be coverage dependent (Vanlerberghe et al. 2011b). The purchase price of the curtains is high, which result in a high strategy cost when implemented by the routine control programme: 1.9 USD per curtain distributed (of which 77% for the curtain itself) (Baly et al. 2011).

Besides being a partial barrier for mosquito entry in houses, the insecticide treated curtains essentially deliver a residual insecticide targeting adult mosquitoes inside the house and may prove an effective alternative to Indoor Residual spraying in targeting adult dengue vectors (Esu et al. 2010).

Perifocal indoor residual spraying has been repeatedly used in Cuba (however few reports of such intervention in other parts of the world are available), but its effect has not been systematically documented. Variable success was observed depending on the insecticide used. In this procedure, a residual formulation of an adulticide is sprayed with a compression sprayer (Hudson type) on the walls of watertanks and its surroundings together with potential *Aedes* resting sites inside the houses (sites where mosquitoes rest after/in between blood meals).

Space spraying with adulticides have been widely and often used in the past by the routine vector control programme of Cuba. From 1981 to 1986 malathion was used as an ultra low volume spray; from 1986 up to present pyrethroids (cypermethrin and Lambda-cyhalothrin) have been used in fogging campaigns during periods of high mosquito infestation levels or during dengue outbreaks (as in Santiago de Cuba in 1997 and 2006 and Havana City 2001-2002). Other products as Bendiocarb have also been occasionally used in the last years (2008 up to now).

Recently, the laboratory of IPK (Institute of Tropical Medicine ‘Pedro Kouri’) evaluated the susceptibility of the 2010 local *Aedes aegypti* strain of Santiago de Cuba. The local strain was found to be resistant to Lambdacyalothrin 0.05%, Bendiocarb 0.1% and DDT 4%. The strain was susceptible for deltamethrin 0.05% and Malathion 5%.

Following these observations, it was decided to compare the deltamethrin treated curtains with peri-focal spraying of deltamethrin (malathion being badly accepted by the population in the past) in Santiago, a municipality where the baseline *Aedes* infestation already reached low levels, but where transmission still occurs.

# III. Research objectives and hypothesis

## *III.A. General Objective*

Compare the acceptance, effectiveness and cost-effectiveness of Insecticide treated curtains (ITC) distribution and of peri-focal insecticide spraying (PFS) to control *Aedes* and dengue.

## *III.B. Specific objectives*

1. Evaluate the uptake and acceptability of tools in both intervention arms (ITC and PFS)
2. Evaluate the effectiveness of both tools, on top of the routine, in the control of *Aedes aegypti* (in comparison to the effect of the routine programme alone)
3. Compare the cost-effectiveness of both tools with the routine *Aedes* control strategy

# IV. Methodology

## *IV.A. Description of study* **and study areas**

The study is planned to take place in urban Santiago de Cuba. Santiago is one of the Cuban municipalities where *Aedes aegypti* foci are most frequently reported, despite continuous surveillance and vector control activities. In 1981 it was affected by an epidemic that spread across the country and in 1997, 2001, 2002, 2006 and 2008 by epidemic outbreaks that remained rather limited. Santiago de Cuba is located in the Eastern part of the island, in the province of the same name. The average temperature oscillates between 30 and 32 °C and rainfall is scarce. The municipality is the most densely populated one of the whole country and has 475 580 inhabitants, of which eighty-seven percent live in the urban zone. There are residential settlements under construction and the periurban neighbourhoods lack adequate sanitary conditions. Water is supplied, on average, every 3 days, but since there were in the last decades major damages to the piped water system, the population is used to store drinking water at home in multiple containers. Garbage collection is sometimes deficient, which leads to accumulation of trash.

Design:

Cluster-randomized controlled trial with 2 years follow up of interventions. Intervention and control clusters will be selected out of all urban municipalities (except Fran Pais and Julian Grimau).

Cluster = a house block with its 4 surrounding house blocks; containing approx. a total of 250 houses

Clusters with the highest infestation – levels (entomological information from January to December 2009) will be selected. The selection procedure will assure that there are no common boundaries between clusters, as a certain spill-over effect can be expected (Kroeger et al. 2006). Afterwards, clusters will be randomly allocated to the control, ITC or PFS intervention arm.

Sample size estimation (based on the effectiveness of curtains, measured through entomological indices):

The Breteau index (BI) was on average 2 per 100 houses in 2009. With the interventions, we aim to decrease the BI with 50% to 1/100 houses. Based on the Hayes and Bennett sample size estimation (Hayes & Bennett 1999), using an inter-cluster variation coefficient of 0.5, we need a minimum of 20 clusters of 250 houses in each arm to demonstrate the effect of the ITC or PFS intervention in comparison to the control arm. We increased the number of clusters with 5% and hence will have a final sample size of 21 clusters of 250 houses/study arm. Totaling 63 clusters of on average 250 houses for the entire study.

## *IV.B. Intervention and routine activities*

To prepare the implementation of interventions, a workshop with the health area responsables for vector control and a workshop with formal and informal community leaders will be held to discuss the detailed implementation planning, as also a meeting with the population of every health area involved to inform them about the trial and answer questions concerning tools and its implementation.

Insecticide treated Curtains (ITC):

The insecticide-treated curtains (ITC) to be used are made from long-lasting, insecticide treated (pyrethroid deltamethrin is applied during manufacture) polyester netting that requires no re-impregnation (PermaNet®; Vestergaard-Frandsen company). PermaNet materials are special UV protected and retain their insecticidal properties and efficacy for about 2 years (information from producer). The material has been approved by WHOPES for use as bednets. ITC will be distributed in each house to be placed in door-openings inside the house or on the closet or on the wall where no or minimal sunshine will touch the material - if a curtain is already hanging, the project-curtain can be attached to it. Per house a maximum of 3 curtains will be distributed, except for schools, working areas and public spaces where more curtains can be distributed.

All curtains will be white patterned netting and have a 1.1m width*2.9m height size (people can adapt them according their needs).

Before distribution the consent of the users will be procured in a community meeting and a written informed consent-form will be obtained (annex 1).

The curtains will be distributed with an accompanying promotion campaign: a Frequently Asked Questions document (FAQ) is already elaborated (annex 2). It will be given to all included households in the ITC arm and will be discussed during distribution on individual basis.

The curtains will be distributed to the houses, but also public places, as working areas, schools, cultural centra will be included as far as possible.

A replacement of curtains will be foreseen for the curtains becoming damaged during use. If new persons are coming to live in the house-blocks during the study, they will be approached to be included in the study.

The procedure of distribution is detailed in annex 3.

Peri-focal insecticide spraying (PFS)

K-Othrine 25 WG, supplied by Bayer Environmental Sciences co. (25% deltamethrin formulation) will be sprayed every 4 months (3 times/year). It is a granular formulation that need to be solved into water (20 gram in 8 Liter of water, sufficient to treat 200 m² and attaining 25 mg a.i./m²). It has a long lasting residual activity. Where deposits remain undisturbed, residual activity depends upon the nature of the surface (Rojas de Arias et al. 2004;Rozilawati et al. 2005). Sustained residual activity beyond 12 weeks post-application is observed on non-porous surfaces. Deltamethrin is photostable and the particulate suspension enhances availability to insects.

The insecticide will be sprayed on the outside of the ground-level water tanks and the walls behind them; and on the adult *Aedes* resting sites (for example under beds, in and under closets) in the intradomestic area.

Teams of health workers of the routine *Aedes* control programme will be trained to realize this activity and standard operating procedures are designed. A short movie has been made to show the correct application of insecticide to all implementers. Control visits by supervisors will be done on a regular basis during application to assure quality of action.

The PFS will be done in the houses, but also public places, as working areas, schools, cultural centra will be included as far as possible.

Before distribution the consent of the users will be procured in a community meeting and a written informed consent will be obtained on the moment of spraying. Information on the product and its possible side effects will be provided to the communities in the preparative community meetings and to each household before spraying starts.

The procedure of implementation is detailed in annex 4.

The set-up of the ITC and PFS interventions will be done by a local research group and by the routine vector control programme. The former will be responsible for its implementation, coordination with the local health authorities, documentation of process and organization of training sessions according to the needs of the actors involved.

Routine *Aedes* Control activities

In all arms, the routine control activities will be continued. In the control-arm, this will be the only strategy. The standard control activities are carried out by the programme’s vector control workers: entomological surveillance and source reduction through periodic inspection of houses, larviciding (with temephos) of water-holding containers, selective adulticiding (cipermethrine and clorpiriphus-fogging) when *Aedes aegypti* foci are detected, providing health education, promotion of community based environmental management and enforcing mosquito control legislation through the use of fines.

## *IV. C. Data collection and analysis*

Effect measures:

Primary outcomes:

Effectiveness: Breteau Index, House index, Pupae per person index, (adult mosquito infestation)

confirmed Dengue cases (if any). Comparison of infestation at each time point between the intervention arms, taking into account the pre-intervention infestation levels and the cluster design. Generalized linear random effect regression models with a negative binomial link function will be used.

Secondary output:

Uptake and use of ITC

Acceptance of PFS and ITC (and identification of underlying lay dimensions of acceptability)

Change in intra-and extradomiciliary risks for Aedes infestation

Cost- effectiveness of ITC and of PFS

Residual insecticidal activity of deltamethrin applied in ITC and in PFS

Data collection:

1. Baseline study (in both intervention and control arms) :
   1. Entomological survey:

Existing of larval and pupal surveys; the larval surveys will be conducted as in the routine system (exhaustive inspection of all houses and potential water holding containers on the presence of immature stages of Aedes). The traditional Stegomyia indices will be calculated: the House Index (HI) and Breteau index (BI). Collection of adult mosquitoes: during routine surveys, the vector control teams are also inspecting houses in the search of adult mosquitoes.

The pupal surveys will consist of direct inspection of potential water-holding containers inside and around the houses following the recommendations of Focks (Focks 2004). This will be done by a special team in 20% of the house-blocks (randomly chosen: 23 houseblocks/arm). All containers where immature stages of Aedes are detected will be emptied and the absolute number of pupae will be counted. For elevated water tanks a sweeping method will be used to collect the majority of the pupae. The total number of positive containers for Ae. aegypti pupae and their relative contribution to total pupal production will be recorded and computed. The number of persons per house will be also enquired (during the distribution of tools) for the construction of Pupal Index per inhabitant (PPI) at the cluster level.

- 1. Evaluation of main intra and extra domiciliary risks at household and environmental risks at community level in each houseblock included in the study: data collected by the routine vector control programme teams through observation, guided by an observation guide (Annex 5).
  2. Evaluation of resistance of the local Aedes strains to the four groups of adulticides commonly used for mosquito control (report, including methodology, in annex 6).

1. Follow-up surveys:
   1. Entomology (in both intervention and control areas):

The same measurements as in the baseline entomological study will be done once intervention started (House Index (HI), Breteau index (BI), Pupal Index per inhabitant (PPI)). The frequency of the larval surveys (including the collection of adult mosquitoes) will be according the cycles taking place by the routine control programme, with at least one survey per month. The pupal surveys will take place twice a year during the peak periods of infestation (end of April and end of September).

- 1. Surveillance of Dengue cases (confirmed) and epidemiology of clinical Dengue cases if an epidemic occurs in the period of study (in both intervention and control areas)
  2. Observation of main intra and extra domiciliary risks at household and environmental risks at community level (data collection as explained above; frequency once/year) – (Annex 5) (in both intervention and control areas)
  3. Uptake and use of curtain (in ITC intervention area)

Uptake and continued use will be evaluated every 4 months over a period of 2 years. The teams will use an observation guide during the household visits (Observation guide: annex 7).

- 1. Registration of executed vector control activities: besides information available in reports of the *Aedes* control programme, information will be obtained from community perspective (Questionnaire: annex 8) (in both intervention and control areas)

Every 4 months, one in 30 households will be questioned concerning the vector control activities (by community or by routine vector control programme) that were realized in the previous period.

- 1. Evaluation of acceptance, use and satisfaction of specific control measure (in intervention areas):
     1. ITC: through household surveys, 4 , 12, 24 months after distribution (in a sample of 400 houses, systematic random sampling – able to detect a 50% acceptance with 5% precision) (data collection form: annex 9)
     2. PFS: through household surveys, 4 , 12, 24 months after distribution (in a sample of 400 houses, systematic random sampling – able to detect a 50% acceptance with 5% precision) (data collection form: annex 10)
  2. Residual activity/bio-availability testing (in intervention areas):
     1. ITC (monitoring insecticide content of PermaNet curtains over time): These bioassays will be done using two methods of exposure: cone method and tube method. In both, the same procedure will be followed: 10 cones/tubes per curtain, 5 female *Aedes* mosquitoes per cone/tube, 3-5 days old, non-blood fed, exposure of 3 minutes, evaluation of knock down after 3’ and 1 hr and mortality after 24 hr. This will be done at 4 time moments (or shorter if decline of residual effect): on a sample of 2 curtains before distribution (non-used ITC to evaluate batch quality), on a sub-sample of 10 curtains, after 6, 12, 18 and 24 months of use. In the 6 month assay, ITC will be collected in 10 houses of vector control workers (where washing procedures and washing frequency and other characteristics of use will be recorded in a prospective way). In the surveys of 12 and 24 months, a random sampling will be done. Additionally a subsample of the curtains (one ITC which gives mortality below 80%, one with mortality between 80-95% and one with mortality > 95%) will be evaluated through HPLC to define the residual deltamethrin concentration (Laboratorio de registro de plaguicidas en Cuba, a certified center in Havana).
     2. PFS:
        - Bio-assays (cone method, with 15 female *Aedes* mosquitoes/cone; 3-5 days old, non-blood fed, exposure of 1 hour – transference to clean cups, observation of knock down/mortality after 1 hour and after 24 hours)
        - Selection of houses: 4 houses (one per health district) with previous indication of surfaces to be tested; and 4 houses (randomly chosen, one per district) with no previous indication of surfaces to be tested
        - Test of : 2 cones on metal surface; 2 cones on wooden surface, 2 cones on cement surface and 2 cones as control on a non-sprayed surface.
        - Frequency: 1 day, 1 week, 2 weeks, 3 weeks, 4 weeks, 2 months, 3 months and 4 months after spraying (or stopped earlier if no mosquito mortality is observed). This will be done after the first application and will be repeated in the second year of application.
  3. Resistance testing of the local Aedes aegypti strains to deltamethrin and other insectcides (as done in baseline) using the WHO standardised impregnated papers (see annex 6 for methodology). Evaluation with one year intervals (starting from baseline): mid 2011, mid 2012 and in 2013 when study finished.

1. Cost-effectiveness evaluation

We will collect during the assay the financial cost of the routine vertical programme and will calculate the average cost per house inspected and treated.

For each arm, we will multiply the number of houses inspected and treated annually during the study by the corresponding average cost described above, to calculate the total annual cost of the routine programme per arm.

We will calculate the total economic cost of the new control tools (ITC and PFS): The economic cost of the community participating in the set up, the financial and economic cost of the routine programme during set up, distribution, installation and maintenance of the tools; and the economic cost of the community participating in collective vector control activities.

The total annual cost of the different arms can be obtained adding the cost of the routine programme and the economic cost of the implemented strategy.

We also will calculate the average and marginal cost per house within arms and incremental cost per house between arms.

The activities of vertical programme will be costed prospectively every month during two years of the study, as well as the cost regarding the distribution of the tools itself, every time it happens (see annex 11-12-13-14-15-16). The cost information about participation in individual and collective activities related to vector control of the community will be followed by questions included in the acceptability survey 3 times during follow up period, in 400 houses per arm for the two intervention arms and with quarterly surveys on the routine activities done (annex 8 ).

We will obtain the effectiveness measure from the entomological and epidemiological study and use the difference of differences between the baseline data and the data for the subsequent periods as effectiveness measure.

We will calculate arithmetic means and 95% confidence intervals when making cost and effectiveness (entomological indices) comparisons between the three arms of the trial. We will carry out incremental analysis, with the mean cost difference between study’s arms divided by corresponding mean difference of entomological indices to give the incremental cost effectiveness ratio (ICER). We will use the non-parametric percentile method for calculating the confidence interval around this ratio with 1000 bootstrap estimates of the mean cost and entomological indices differences. We will carry out sensitivity analysis, varying the price of the tools, and using the variation observed in the correlation between the coverage of tools and the corresponding entomological outcome.

1. Sociological qualitative study on acceptability

In-depth interviews will be conducted in a purposive sample of 20 households where ITC have been distributed and PFS initiated. The households will be identified from data collected by the household survey and through program staff. The interviews will be conducted:

- - 1. 4 months after distribution of ITC and PFS with 10 household heads which have either 1°) not accepted the tools at the onset; 2°) accepted the tools or 3°) accepted with reluctance.
    2. 12 months after distribution of ITC and PFS with 10 household heads which have either 1°) accepted the tools, 2°) accepted with reluctance, and 3°) who have stopped to use them after a period of one year.

The sampling is tentative and could be increased depending on the results of preliminary data analysis. We will also explore the perceptions of the interviewees on the routine *Aedes* *aegypti* control strategy (topics guide and informed consent for in-depth interview: annex 17 and 18).

## *IV.D. Quality Control*

1. Documentation and standardization of the different activities to be executed in the project:
   1. Standardization of training, repeated in each health area
   2. Description of process of PFS (annex 4) and of ITC implementation (annex 3)
   3. Data collection for pupal surveys
   4. Data collection for entomological surveys (existing routine system is standardized); quality control routinely done in 60% of the households
2. Data entry will be performed in Access, questionnaires already coded, a control check of 5% of the data will be done by an external independent person
3. The information given in questionnaires will be cross-checked with direct observation if indicated.

## *IV. E. Ethical concerns*

The protocol will be revised by the ethical committee of the Institute of Tropical Institute (IPK), Havana and the Cuban health authorities, by the scientific board of Institute of Tropical Medicine, Antwerp, Belgium, and the ethical committee of the University of Antwerp, Belgium.

Informed consent from local authorities and community representatives for distributing new vector control tools in the area will be obtained. Informed consent of interviewees and Focus group discussion participants will be taken. The researcher will explain the research project: the objectives, the funding, methods, the involvement of the participants and the length of time of involvement, and plan on the use of the research outcomes, including how these will be disseminated. All this information will be placed in the written Informed Consent form for the ITC areas (annex 1).

For the houses included in the PFS arm, no special informed consent form was designed (as residual spraying is not a new activity and has already been implemented by the routine programme), the signature of informed consent will be obtained on the form filled in by the health worker during application.

The confidentiality of data will be ensured during data management and analysis. Participants will not be identified in any written or oral report, especially in relation to the data they provided.

**Critical assessment and possible limitation of the study design and methodology in relation to project objectives:**

The main limitation is the use of the routine entomological information to compare the effectiveness between study groups: this data collection has standardized procedures. However different vector control technicians are collecting it and few data can be (randomly) missing. We will improve the quality assurance system in place to secure validity of the measurement of entomological variables.

## Timeline

|  | Year 1 |  |  |  |  |  |  |  |  |  |  |  | Year 2 |  |  |  |  |  |  |  |  |  |  |  | Year 3 |  |  |  |  |  |
| --- | --- | --- | --- | --- | --- | --- | --- | --- | --- | --- | --- | --- | --- | --- | --- | --- | --- | --- | --- | --- | --- | --- | --- | --- | --- | --- | --- | --- | --- | --- |
|  | 1 | 2 | 3 | 4 | 5 | 6 | 7 | 8 | 9 | 10 | 11 | 12 | 1 | 2 | 3 | 4 | 5 | 6 | 7 | 8 | 9 | 10 | 11 | 12 | 1 | 2 | 3 | 4 | 5 | 6 |
| Preparation and baseline | X | X | X |  |  |  |  |  |  |  |  |  |  |  |  |  |  |  |  |  |  |  |  |  |  |  |  |  |  |  |
| Distribution of curtains |  |  |  | X |  |  |  |  |  |  |  |  |  |  |  |  |  |  |  |  |  |  |  |  |  |  |  |  |  |  |
| Perifocal spraying |  |  |  | X |  |  |  |  | X |  |  |  | X |  |  |  | X |  |  |  | X |  |  |  | X |  |  |  |  |  |
| Entomological evaluation |  |  |  | X | X | X | X | X | X | X | X | X | X | X | X | X | X | X | X | X | X | X | X | X | X | X | X | X |  |  |
| Pupal surveys |  |  |  | X |  |  |  |  | X |  |  |  |  |  |  | X |  |  |  |  | X |  |  |  |  |  |  | X |  |  |
| Acceptance evaluation |  |  |  |  |  |  |  |  | X |  |  |  |  |  |  | X |  |  |  |  |  |  |  |  |  |  |  | X |  |  |
| ITC Use survey |  |  |  |  |  |  |  |  |  |  |  |  | X |  |  |  |  |  |  |  | X |  |  |  | X |  |  |  |  |  |
| Environmental risks |  |  | X |  |  |  |  |  |  |  |  |  |  |  | X |  |  |  |  |  |  |  |  |  |  |  | X |  |  |  |
| Registration VC activities |  |  |  |  |  | X |  |  |  | X |  |  |  | X |  |  |  | X |  |  |  | X |  |  |  | X |  |  |  |  |
| Residual activity testing ITC |  |  |  | X |  |  |  |  |  | X |  |  |  |  |  | X |  |  |  |  |  | X |  |  |  |  |  | X |  |  |
| Residual activity testing PFS |  |  |  | X | X | X | X |  |  |  |  |  |  |  |  |  |  |  |  |  | X | X | X | X |  |  |  |  |  |  |
| Susceptibility testing |  |  |  |  |  |  | X | X | X |  |  |  |  |  |  |  |  |  | X | X | X |  |  |  |  |  |  |  | X | X |
| Economic evaluation |  |  |  | X | X | X | X | X | X | X | X | X | X | X | X | X | X | X | X | X | X | X | X | X | X | X | X | X |  |  |
| Analysis and report writing |  |  |  |  |  |  |  |  |  |  |  |  |  |  |  |  | X |  |  |  |  |  |  |  |  |  |  | X | X | X |

Reference List

1. Baly A, Flessa S, Cote M *et al.* (2011) The Cost of Routine Aedes aegypti Control and of Insecticide-Treated Curtain Implementation.*Am.J.Trop.Med.Hyg.* **84**, p 747-752.

2. Esu E, Lenhart A, Smith L, & Horstick O (2010) Effectiveness of peridomestic space spraying with insecticide on dengue transmission; systematic review.*Trop.Med.Int.Health* **15**, p 619-631.

3. Focks, D. A review of entomological sampling mlethods and indicators for dengue vectors. WHO/TDR/IDE/Den.03.1. 2004.

4. Hayes RJ & Bennett S (1999) Simple sample size calculation for cluster-randomized trials.*Int.J.Epidemiol.* **28**, p 319-326.

5. Kroeger A, Lenhart A, Ochoa M *et al.* (2006) Effective control of dengue vectors with curtains and water container covers treated with insecticide in Mexico and Venezuela: cluster randomised trials.*BMJ* **332**, p 1247-1252.

6. PAHO. Dengue and dengue haemorrhagic fever in the Americas: guidelines for prevention and control. Sci. Pub 548. 1994. Washington DC, Pan American Health Organization.

7. Rojas de Arias A, Lehane MJ, Schofield CJ, & Maldonado M (2004) Pyrethroid insecticide evaluation on different house structures in a Chagas disease endemic area of the Paraguayan Chaco.*Mem.Inst.Oswaldo Cruz* **99**, p 657-662.

8. Rozilawati H, Lee HL, Mohd MS, Mohd N, I, & Rosman S (2005) Field bioefficacy of deltamethrin residual spraying against dengue vectors.*Trop.Biomed.* **22**, p 143-148.

9. Vanlerberghe V, Villegas E, Jirarojwatana S *et al.* (2011a) Determinants of uptake, short-term and continued use of insecticide-treated curtains and jar covers for dengue control.*Trop.Med.Int.Health*.

10. Vanlerberghe V, Villegas E, Oviedo M *et al.* (2011b) Evaluation of the effectiveness of insecticide treated materials for household level dengue vector control.*PLoS.Negl.Trop.Dis.* **5**, p e994.

11. WHO HQ, Geneva. Strengthening Implementation of the Global Strategy for Dengue Fever/Dengue Haemorrhagic Fever Prevention and Control. Report of the Informal Consultation 18-20 October 1999. (WHO/CDS/(DEN)/IC/2000.1). 2000.
